# Eosin Removal by Cetyl Trimethylammonium-Cloisites: Influence of the Surfactant Solution Type and Regeneration Properties

**DOI:** 10.3390/molecules24163015

**Published:** 2019-08-20

**Authors:** Fethi Kooli, Souad Rakass, Yan Liu, Mostafa Abboudi, Hicham Oudghiri Hassani, Sheikh Muhammad Ibrahim, Fahd Al Wadaani, Rawan Al-Faze

**Affiliations:** 1 Al-Mahd Branch Community College, Taibah University, Al-Mahd 42112, Saudi Arabia; 2 Department of Chemistry, Taibah University, P.O. Box 30002, Al-Madinah Al-Munawwarah 41147, Saudi Arabia; 3 Institute of Chemical and Engineering Sciences, 1 Pesek Road, Jurong Island, Singapore 627833, Singapore; 4 Engineering Laboratory of Organometallic and Molecular Materials, Chemistry Department, Faculty of Sciences, University Sidi Mohamed Ben Abdellah, P.O. Box 1796 (Atlas), Fez 30000, Morocco; 5 Department of Chemistry, Faculty of Science, Islamic University of Madinah, Al-Madinah Al-Munawwarah 42351, Saudi Arabia

**Keywords:** clay minerals, organophilic clays, removal, eosin dye, regeneration

## Abstract

The effect of the counteranion of hexadecyltrimethylammonium salts on the physico-chemical properties of organoclays was investigated, using a selected natural clay mineral with a cation exchange capacity of 95 meq/100 g. The uptake amount of C_16_ cations was dependent on the hexadecyltrimethylammonium (C_16_) salt solution used, the organoclay prepared from C_16_Br salt solution exhibited a value of 1. 05 mmole/g higher than those prepared from C_16_Cl and C_16_OH salt solutions. The basal spacing of these organoclays was in the range of 1.81 nm to 2.10 nm, indicating a similar orientation of the intercalated surfactants, and could indicated that the excess amount of surfactants, above the cation exchange capacity of 0.95 meq/g could be adsorbed on the external surface of the clay mineral sheets. These organoclays were found to be stable in neutral, acidic, and basic media. The thermal stability of these organoclays was carried out using thermogravimetric analysis and in-situ X-ray diffraction (XRD) techniques. The decomposition of the surfactant occurred at a maximum temperature of 240 °C, accompanied with a decrease of the basal spacing value close to 1.42 nm. The application of these organoclays was investigated to remove an acidic dye, eosin. The removal amount was related to the initial used concentrations, the amount of the surfactants contents, and to the preheated temperatures of the organoclays. The removal was found to be endothermic process with a maximum amount of 55 mg of eosin/g of organoclay. The value decreased to 25 mg/g, when the intercalated surfactants were decomposed. The reuse of these organoclays was limited to four regeneration recycles with a reduction of 20 to 30%. However, noticeable reduction between 35% to 50% of the initial efficiency, was achieved after the fifth cycle, depending of the used organoclays.

## 1. Introduction

The demand for water, especially fresh water for the comfort of human society, is sharply increasing due to the rapid increase in population and industrial activities [1,2,3]. Some industrial sectors use tremendous amounts of water in their daily activities, leading to generation of huge amount of wastewaters polluted with different kinds of pollutants, among them inorganic and organic materials such as heavy metals, dyes, aromatic, and phenolic compounds, depending on the type of their activities [4]. During the years, the use of dyes has increased and nowadays immense amounts are used in various sectors like the textile, pulp and paper, paint, pharmaceutical, cosmetics, food, printing inductries, etc. [5,6]. The discharge from these industries is highly colored, as enormous amount of dyes remain unfixed (reaching up to 50%) during coloring and washing, and are discharged in the effluents [7]. The dye effluent if discharged untreated can affect the photosynthesis of aquatic plants and thus, the oxygen levels necessary for the survival of the aquatic fauna and flora [8,9].

To solve this problem, the reclamation, recycling, and reuse of wastewater were proposed to meet the water requirements for industry and irrigation uses, where 75% of total consumption can be attributed to agriculture [10]. Different techniques are proposed to treat dye-contaminated wastewaters, and generally different factors must be considered such as safety, efficiency and budget. The merits and demerits of each of these techniques were presented in a review by Ashfaq and Khatoon [11].

Currently, the removal of dyes by adsorption techniques is proved to be an effective and attractive process for the treatment of dye-contaminated effluents [11,12]. This method is simple and easy to operate, and it has an edge over other methods due to its sludge-free clean operation and complete removal of dyes, even from dilute solutions [13,14]. Activated carbon is the most widely used in this process due to its promising physico-chemical properties such as extended surface area, microporous structure, and exceptional surface reactivity. However, the high cost and regeneration difficulties have increased the need to explore lower cost and reusable materials [15,16]. Many cheaper adsorbents were proposed by different researchers, ranging from natural to synthetic materials, and many reviews were published regarding these materials [17,18,19,20]. Among the natural materials, clay minerals were proposed as good candidates due to their outstanding adsorption properties [21,22]. However, these clay minerals are typically subjected to many modifications prior their use, depending of the target pollutant and its charge [23,24]. The clay minerals are efficient at removing cationic metals and positively charged organic pollutants such as basis dyes [23], due to their negatively charged and hydrophilic surface, however, in case of nonpolar hydrophobic contaminants or negatively charged pollutants such as acid dyes or anions, clays are largely ineffective due to the electrostatic repulsion; and their hydrophilic character as mentioned above [25,26]. Thus adequate modifications are generally proposed. The most common modification consists of the use of cationic surfactant solutions that generate organoclay adsorbents that combine both the properties of the inorganic layered material and hydrophobic environment with the intercalation of the organic cations. Indeed, these materials adsorb a large range of pollutants, such as pesticides [27], phenolic compounds [28], various pharmaceutical products [29] and acidic dyes [23,24]. The removal efficiency of these organoclays mainly depends on both the chemical nature and the structural organization of the intercalated surfactants [30,31]. Thus, surfactants possessing long alkyl chains such as hexadecyltrimethylammonium (C_16_) create an appropriate organic environment within the inorganic frame for the adsorption of alkanes and aromatic compounds [29]. Based on the studies of Ma et al., where the removal properties of organoclays were closely related to the length of the alkyl chains, numerous studies were devoted to modify the raw clay minerals by longer alkyl chains such as hexadecyltrimethylammonium cations [32]. The density of C_16_ surfactants in the interlayer spacing affects the removal properties. A maximum amount equivalent to 1 time the cation exchange capacity (CEC) of the clay mineral allows the creation of a hydrophobic environment without any strong steric effects that may restrict the removal of pollutants, while the uptake of surfactants at high concentration (i.e., > 1 CEC) creates a large hydrophobic network with an arrangement of the organic cations in the bilayer within the interlayer space that may enhance or reduce the removal properties [29]. The removal of acidic dyes was improved when the content of C_16_ cations was increased and exceeded the CEC values, due to favourable interactions arising with the R groups of the quaternary ammonium ions [24,33], which made the acidic dyes more easily attracted into the expanded interlayer space of the clay [33].

The properties of organoclays strongly depend on the structure and the molecular arrangement of the organic surfactants within the interlayer spacing of the clay minerals [34,35]. However, in other cases, the origin of clay minerals and the type of C_16_ salt used to modify the clay mineral was crucial and it has to be taken in consideration during the preparation of the organoclays [33,36,37]. The commonly used C_16_ modifiers are prepared by dissolving their solid salt with bromide anion in water solutions [24,33]. Few studies using chloride or hydroxide C_16_TAM salts are reported [33,36,38,39,40,41,42]. The type of anion was found to affect the critical micelle concentration (cmc) of the surfactant solution, for instance, C_16_Cl solution has a cmc value of 1.4 mM, higher than the cmc of C_16_Br (0.94 mM) in deionized water, due to the smaller size of the Cl^−^counterion compared to the Br^−^ counterion. [43,44], and the shape of the micelles. Indeed, the micellar form changes from globular to highly elongated for C_16_Br solutions, while, the micelles from C_16_Cl solution tend to remain nearly spherical over all the concentrations range [45]. The C_16_Br salt led to the highest intercalated amount, resulting to an organoclay with a basal spacing varying from 3.80 nm to 4.1 nm, and with good removal capacity of the acidic dye eosin, using a polymer grade montmorillonite [33]. For other modifications, the origin of the clay mineral has an interesting effect of the properties of resulting modified clays [46,47]. For example, in case of acid activation processes, two clay minerals from different origins were examined and they found to behave differently towards the acid activation process, thus leading to materials with different C_16_ cation uptake properties [46,47]. The removal of a basic dye from an aqueous solution was thus also dependent on the origin of the clay used to prepare the absorbent [48].

This study was carried out to test the hypothesis that the nature of the C_16_ solutions (in term of their anions) might affect the uptake amount of C_16_ cations for another type of clay mineral, thus, leading to organoclays with different physiochemical properties, and determine if the resulting materials would be effective in the removal of the acidic dye eosin. The thermal stability of organoclays was examined using in-situ x-ray diffraction (means at real temperatures during the collection of the patterns, without cooling down the samples). This part will provide an idea how the pre-heated oranoclays might affect the removal properties of the eosin dye. Eosin was chosen as model pollutant because it has been known for a long time, and it has a variety of usages, mainly as a biological stain, as a pH indicator, and a dye in the wool, silk, modified acrylic, cosmetic and pharmaceutical industries, etc. [49,50,51,52].

Different techniques were used to characterize the organoclay materials prior their application in the removal of a selected anionic dye, eosin. Regeneration tests were carried out to study further valorization of these organoclays, and an economical and friendly method was used in this regard [33,48].

## 2. Results and Discussion

### 2.1. Characterization of Organoclays

#### 2.1.1. Elemental Analysis

The CHN elemental provides an estimation of the uptake of organic cations in the prepared organoclays [33,53,54,55,56,57]. The data are summarized in Table 1. For an initial loading concentration of 2.40 mmole (corresponded to an initial CEC/mmole of C_16_ ratio of 2.95), the highest content (28.12%) was achieved when C_16_Br solution was used, and the lowest one (18.90%) was attained starting from C_16_OH solution. Using a chloride solution, a value of 20.47% was obtained. Hence, the organic carbon in the organoclays was almost entirely derived from the exchanged organic cations.

The ratio of C/N for modified-cloisites, obtained from elemental analysis data, was 18.35 to 18.89, the calculated value of the C/N ratio for C_16_ was 18.87 close to the theoretical value of 19. This value was higher than the reported value of 16.29 [58]. These results confirm that the modification of cloisite clay mineral was successfully achieved with C_16_ cations [58].

The uptake C_16_ cations was close to the CEC value, as indicated by the ratios (uptake amount/CEC) values of 1, using C_16_Cl or OH solutions. However, it was higher than the CEC, when the organoclay was prepared from C_16_Br solution, with a ratio value of 1.44. Similar observations were reported when using different clay minerals [33]. Attempts have been carried out to tune the content of C_16_^+^ cations in chloride solutions, by varying the initial loading concentrations from 0.2 mM to 2.4 mM. The uptake was improved by increasing concentration of the loaded C_16_Cl solutions from 0.22 mmole/g to 0.90 mmole/g, and it was slightly affected by further increase of the C_16_Cl concentrations, and a maximum uptake of 1.04 mmol/g was achieved (Table 2). Similar data were obtained when C_16_OH solution was used, for another type of clay mineral with low cation exchange capacity (CEC) [59].

The obtained data indicated that the uptake of C_16_ cations followed two trends; the first one was below or close to the cation exchange capacity (CEC) values, and the second one was higher than CEC values. The first trend confirmed that the uptake of C_16_ cations occurred mainly via cation exchange reactions, where the exchangeable Na cations were replaced by C_16_ ones, when C_16_OH or C_16_Cl solutions were used [33]. An additional mechanism took place when a C_16_Br solution atinitial loading concentrations higher than 1.5 mmole/g was used. It could be related to the hydrophobic bonding which includes the mutual attraction between the alkyl chains of surfactant molecules, or to the formation of organocation aggregates on the used clay surfaces [59,60,61].

**Figure 1:**
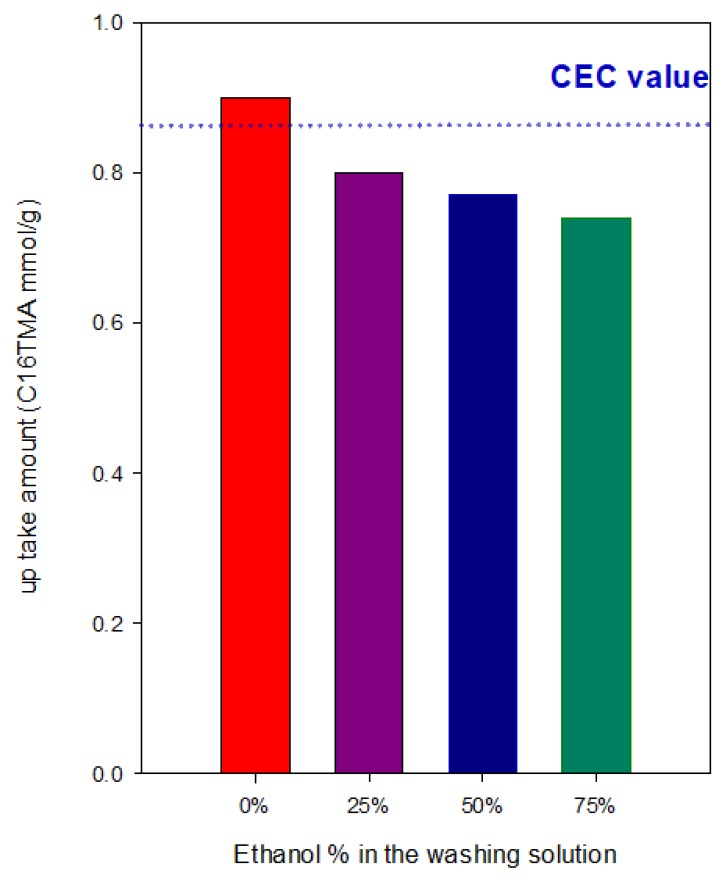
Effect of the ethanol content in the washing solution of the organoclay.

The reported mechanism could be modified by the type of washing solution used, as reported in previous studies [33]. In the present case, the C_16_Cl-CN-2.4 sample was prepared in pure deoinized water, however it was washed with different mixtures of ethanol and water (in volume). Figure 1 indicates that the content of up take C_16_ cations was slightly modified even by using a percentage of ethanol to water of 75%, it varied from 0.82 mmole/g to 0.75 mmole/g. Different results were reported when the uptake amount exceeded largely the CEC values and using different C1_6_Br solutions [33]. It was reported that the addition of alcohols into C_16_Br solutions considerably affected the critical micelle concentration (CMC) value and the degree of counterions bound to its micelles [62,63,64], in other words, the CMC of C_16_Br solution increased with the increasing content of alcohol in the solution because of the stronger interactions of C_16_Br hydrophobic tail with ethanol than with water which made micellization more difficult. Moreover, ethanol molecules act as solvent structure modifiers, reducing the hydrophobic effect in the solution and therefore increasing the cmc [62,63].

The CHN analysis indicated that the C_16_ClCN-2.4 material was stable, after treatment of the solid organo-clay in different solutions of NaCl, NaOH and HCl. These data implied that intercalated C_16_ cations were difficult to exchange either by Na cations or by protons [64,65]. These data were different when the intercalated cations were located between other layered silicates such as magadiite and kenyates [54,65]. This difference could be related to the different orientations of the intercalated surfactant cations in the interlayer spacing.

#### 2.1.2. Powder XRD Data

**Figure 2:**
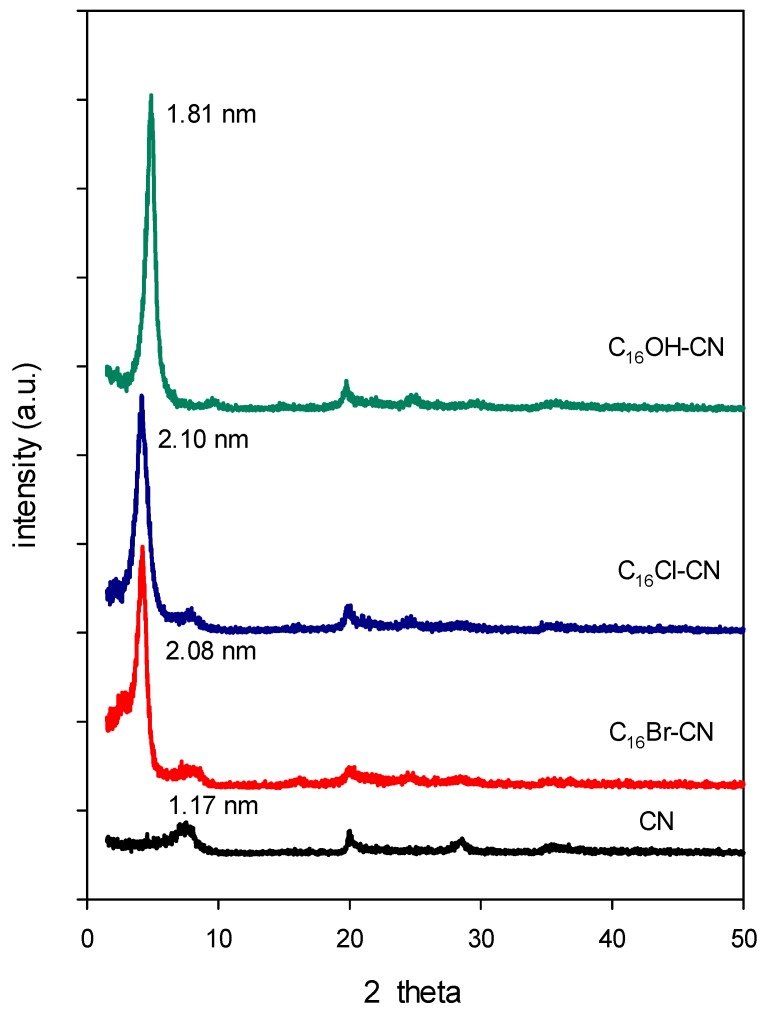
Powder XRD patterns of cloisite clay (CN) exchanged with different C_16_ solutions.

Because of its easiness and its availability, XRD is most commonly used to probe the success of cloisite modification and its structure, by monitoring the position of the basal reflections. The obtained data are presented in Figure 2. The cloisite clay exhibited a basal distance of 1.17 nm, assigned to the (001) basal spacing of the clay, as reported by Bertuoli et al. [66]. After reaction with C_16_ surfactant solutions at a fixed loading, the position of the first reflection (001) of the resulting organoclays shifted to lower 2θ angular values, corresponded to an expansion of the basal spacing, varying from 1.81 nm to 2.10 nm. These data confirmed the success of the modification of the raw cloisite, and the intercalation of surfactants between the starting cloisite sheets [67].

The basal spacing values were close to each other, by taking in account the error of measurement, and were independent of the C_16_ solutions used, and it could indicate that the type of solution used had no effect on the intercalated amounts and thus the orientation of the C_16_ cations within the basal spacing, in good agreement with the CHN analysis. However, using a different clay from another source, different basal spacings were obtained in the range of 2.00 nm to 4.20 nm [33].

The measured value for C_16_Cl-CN clay was close to that reported for other organoclays using the same C_16_ solutions [36]. Attempts have been carried out to increase the basal spacing by varying the initial loading concentrations of C_16_Cl solution. An average expansion the basal spacing of 2.00 nm was obtained for initial loading greater than 0.6 mM (Supplementary Material 1). These data were different when using another starting clay from a different source, as reported in previous study [33].

The lack of variation of the basal spacing indicated that the C_16_ contents did not have an effect on the basal spacing expansion, and the uptake amount that exceeded the CEC value could be adsorbed on the external surface of the clay sheets.

Similar results were obtained using C_16_OH solution at different initial concentrations. In this case, the basal spacing was independent of the used concentrations greater than 0.60 mM. An average of basal spacing of 1.89 nm was obtained. Similar data were obtained using clays from different sources, and it could be related to the chemistry of C_16_OH solution [33,68]. However, in a particular case when a high loading concentration was used at above 25 mmole, further extension of the basal spacing was achieved and reached a maximum of 3.56 nm [68].

When a mixture of water and ethanol was used to wash the organoclay, the basal spacing of the organoclay of 2.02 nm was not affected and it remained unchanged, in good agreement with the CHN analysis, and with other reported studies for organoclays with the same basal spacing of 2.02 nm. However, in case of higher expansion value of 3.91 nm, the effect of the ethanol-water mixture was noticed and led to a shrinkage of the basal spacing at 2.02 nm, either by using as a medium reaction or washing solutions [33]. However, Gates showed that clays exchanged with a substituted alkylammonium cations swelled substantially in mixed solutions of ethanol-water, which was not the present case [64].

The PXRD data indicated that no variation of the basal spacing at 2.02 nm was observed after a contact of overnight within different environments such neutral, base or acid solutions. Owing that the intercalated C_16_ cations were difficult to exchange with smaller cations such Na cations and protons, and might indicate that the intercalated organic cations were strongly attached to the clay layers. Different results were obtained from an organo-magadiites or organo-kenyaites [53,65] with different silicate layered structures.

**Figure 3:**
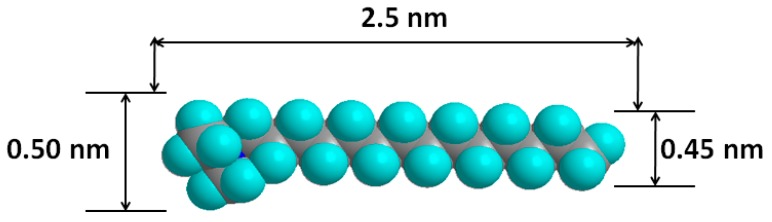
Model structure of the C_16_ cation and its dimensions.

C_16_ cations have a 16-carbon as a long tail and an ammonium head group with three methyl groups attached. It has a length is about 2.3–2.5 nm and a thickness of 0.50 to 0.45 nm [69], as presented in Figure 3.

Theoretical calculations could be used to predict and to confirm the results about intercalation of organic cations in clay minerals. They were based on the length of the main carbon chain of the compound and the basal spacing of the unmodified clay mineral. Equation (1) can be used to calculate the theoretical basal spacing of an organoclay.

d(001) = k(n − 1) + d_c_ + d_m_ (1)

where: n = number of carbon atoms in the surfactant chain, d_c_ = basal spacing of the unmodified clay mineral, d_m_ = the van der Waals radius of the terminal methyl group (0.4 nm), k (constant) = 0.126 (calculated from the increase, in length, for each C-C bond in the chain). This equation assumes that the alkyl group of the organic cations adopts a totally extended molecular conformation or a *trans-trans* chain conformation perpendicular to the clay surface [70].

The theoretical basal spacing is expected to be at 3.25 nm, the experimental basal spacing values (of 1.88 to 2.02 nm) were lower than the theoretical one, suggesting that the chains of C_16_ cations exhibited a bilayer of flat surfactants lying parallel the clay layers, instead of being perpendicular to the surface as assumed by the theoretical model. These data were in good agreement with those reported for similar basal spacing of C_16_ organoclays.

**Figure 4:**
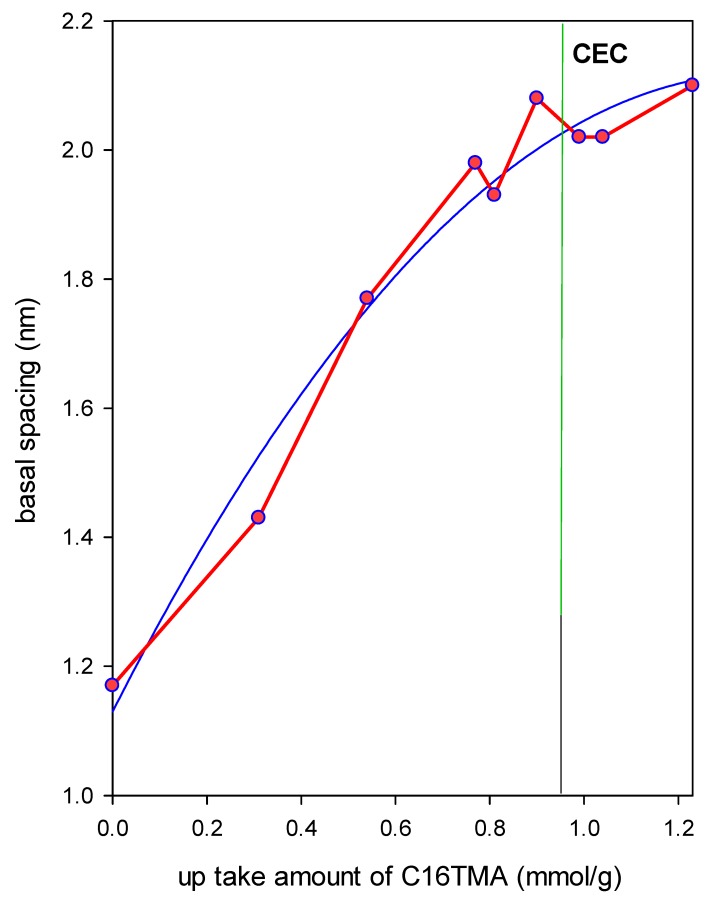
Variation of the basal spacing expansion with the up take amount of the surfactants (mmole/g, red line), blue line corresponds to the fitting curve.

Attempts were made to relate the expansion of the basal spacing with the up take amount of C_16_ cations, and the data confirmed that the up take amount exceeding the CEC value was indeed adsorbed onto the surface of clay layers and they were not located in the interlayer spacing (Figure 4). The data fitted well the best Equation (2):

d(nm) = 1.129 + 1.439U − 0.523U^2^ (2)

with a regression coefficient r^2^ of 0.9730, where U represents the uptake of C_16_ cations (mmole/g).

#### 2.1.3. FTIR Data

**Figure 5:**
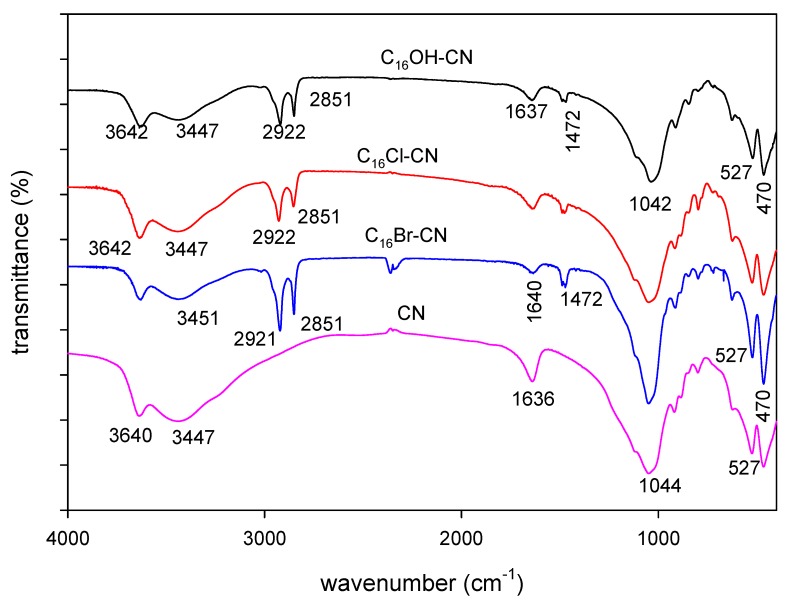
FTIR spectra of starting cloisite clay (CN) exchanged with different C_16_ solutions.

The FTIR technique was employed to confirm the presence of the C_16_ cations and their conformation between the clay layers [71,72,73]. The spectra of pure cloisite and organo-derivatives are presented in Figure 5. The cloisite Na^+^ exhibited bands in the 3100–3700 cm^−1^ range, attributed to the stretching vibrations of the hydroxyl groups bonded to aluminium atoms of the clay mineral [74]. The broad band at 3440 cm^−1^ and a sharp band at 1636 cm^−1^ are assigned to hydroxyl stretching and H-O-H bending vibrations, respectively, of the free and interlayer water molecules in cloisite [75,76]. The band observed at 1040 cm^−1^ was assigned to Si-O stretching mode of the Si–O and Si–O–Si groups, with Si-O and Al-O bending bands at 400–600 cm^−1^. The Mg-O bending band was observed at 470 cm^−1^ [74]. These bands were also observed in FTIR spectra of all modified cloisites, indicating that the structure of the clay layers were not altered. However, a decrease of the bands intensities of 3440 and 1630 cm^−1^ reflects that the amount of hydrogen bonded H_2_O molecules present in the organoclays was less than those with the starting cloisite. This could indicate that the H_2_O content was reduced with the exchange of Na hydrated cations by C_16_ ions (see TGA section), and supported the hydrophobic character of the organoclays (Figure 5) [76].

In addition some new bands were recognized in the FTIR spectra in the range of 2800–3000 cm^−1^which belonged to the organic part of the organoclays. The spectrum of powdered C_16_Br salt (Supplementary Material 2) exhibited the asymmetric and symmetric stretching bands of the N^+^(CH_3_)_3_ group at 3016 and 2945 cm^−1^. When C_16_ cations are intercalated between clay sheets, the motion of the methyl groups are strongly restricted by the strong interactions between the silica framework and the quaternary ammonium moiety. As a result, the asymmetric stretching bands of the N^+^(CH_3_)_3_ group disappeared or decreased in intensity, while the symmetric stretching bands (2959 cm^−1^) were still present [77]. Two intense two bands at 2918 cm^−1^ and 2850 cm^−1^ were assigned to the CH_2_ asymmetric stretching mode (ν_as_ (CH_2_)) and symmetric stretching mode (ν_s_(CH_2_)), respectively [71]. However, in C_16_Br aqueous solution, ν_as_ (CH_2_) shifted from 2919 cm^−1^ to 2932 cm^−1^ and ν_s_ (CH_2_) shifted from 2850 cm^−1^ to 2864 cm^−1^. This shift was related to the changes of conformation of the alkyl chains (Supporting Material 2). In case of organoclays, these two bands were clearly observed at 2921 cm^−1^ and 2851 cm^−1^. Their positions were close to that of C_16_ solid salt, and indicated that the alkyl chains adopted a similar conformation compared to the solid C_16_Br salt.

Previous studies have reported that the position of these two bands was related to the content of the surfactants in the organoclays [76,78,79]. It is well established that the wavenumbers of the CH_2_ stretching bands of hydrocarbon chains are extremely sensitive to the conformational ordering and change in the *gauche-trans* conformer ratio of the chains [71,80] which can be used as probe, in correlation with the d001 spacing variation, for the surfactant arrangement within the silicate layers. Here, the wavenumbers of both symmetric and antisymmetric CH_2_ stretching vibrations indicated that the organic cations located in the internal structure show an all trans conformation. In the present case, it was difficult to observe a change in position, though there was a variation in the surfactant content. This fact could indicate the actual variation in C_16_ cations was not enough to affect the position on these two bands.

The C_16_Br salt exhibited additional bands in the range of 1400–1600 cm^−1^, as a single band, or doublet, or triplet, depending of the origin of the used salt and the technique used to collect the spectrum [73,81]. These bands appeared at 1480, 1473, and 1462 cm^−1^, and were assigned to methylene scissoring mode, or to N^+^-CH_3_ symmetric stretching vibrations. The split of 10 cm^−1^ was due to the intermolecular interaction between two adjacent hydrocarbon chains in a perpendicular orthorhombic subcell [82]. The frequency of these two bands at 1473 and 1462 cm^−1^ in the organoclays are almost independent of the C_16_ cations, and indicated that the intercalated surfactants adopted similar chain conformations, and similar changes in the methyl deformation (Figure 5). The methyl groups were probably linked into the siloxane surface and hence the free rotation of the methyl groups is lost [83], in addition to interchain interaction between contiguous CH_2_ groups of adjoining chains could result in the non-variation of the positions of these bands [84].

#### 2.1.4. Solid ^13^C-CP NMR

**Figure 6:**
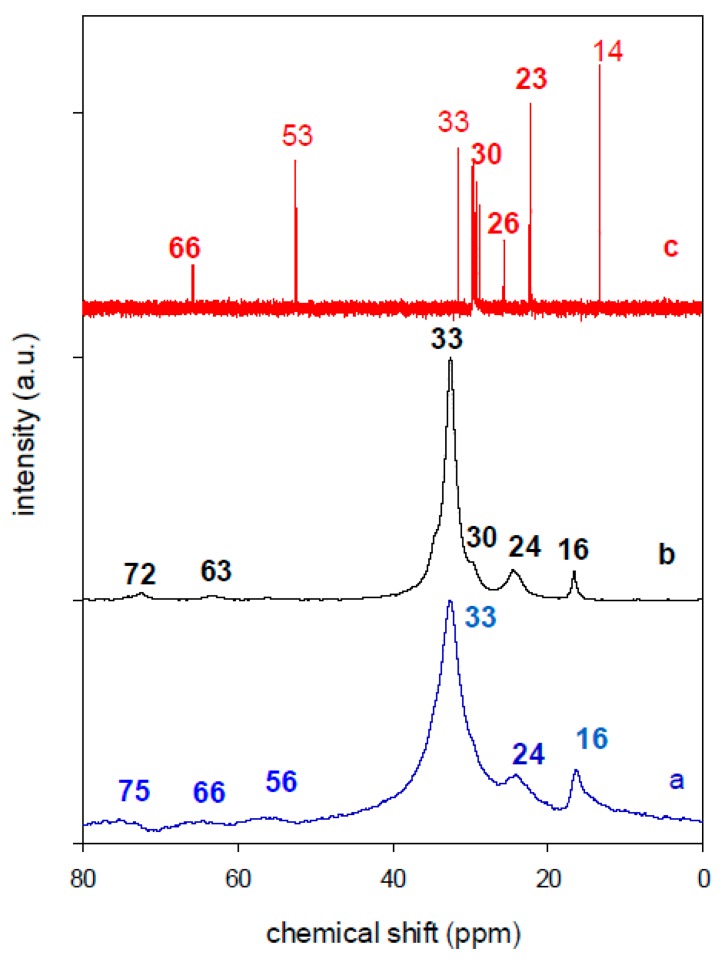
Solid ^13^C-CP-NMR of the (**a**) organoclay C_16_Cl-CN, (**b**) the C_16_Br solid salt, and (**c**) C_16_Br liquid.

The solid ^13^C-CP-NMR technique was used to detect the conformation of the intercalated C_16_ cations in the interlayer spacing of the organoclays [72,85,86]. In general, the ^13^C-CP-NMR spectra were compared to the one of the crystalline salt. In this study, the spectrum of an aqueous solution was also reported for comparison, and they are presented in Figure 6. The chemical shifts of C_16_Br in solution can be easily distinguished and labelled according to the structural formula. The different assignments were related to the numbering of the carbon atoms in the surfactant structure, as presented in Table 3. The extremely narrow peaks for all the carbon groups in the surfactant solution indicate that surfactants are free-moving molecules in a liquid phase (highly mobile). As the mobility and the environment of the carbon atoms in different situations are very influential, broader resonances with slight shifts were observed for C_16_Br in the crystalline powder compared to the solution, which is due to the higher packing density in the solid [87].

The presence of C_16_ cations in the organoclays (for example C_16_Cl-CN) was evidenced by resonance bands summarised in Table 3, with an intense band at 33 ppm, related to internal methylenes (C4-C14). There is a marked difference between the spectra of C_16_-clays and those of the surfactant solution [86]. All peaks for the surfactant solution are much narrower than those for the surfactant-clays. Peaks C3 and C15 are completely resolved from the main C4-C14 peak in surfactant solutions. The electrostatic binding between the surfactant and the clay sheets causes a downfield shift for the methyl groups next to the head group (C1) and substantial broadening for all the peaks [87].

The peak corresponding to the methylene group exhibited the largest broadening among all carbon groups. Upon intercalation, the spectra exhibited broader lines. The line broadening can be attributed to a reduced mobility of the alkyl chain, which in turn should result from a changed hydrocarbon chain packing [88]. The broader and weaker C2peak in the surfactant clays relative to those of other carbon groups indicated that the motion of the surfactant head group adsorbed onto a clay sheets was strongly hindered [89,90].

**Figure 7:**
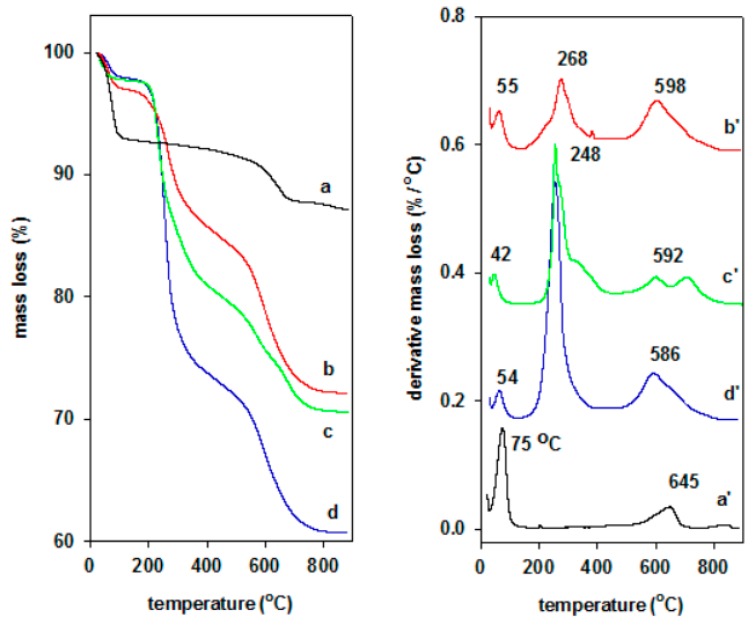
TGA (left) and DTG (right) fetaures of (**a**,**a’**) cloisite raw clay exchanged with different C16 solutions (**b**,**b’**) C16OH, (**c**,**c’**) C16Cl, and (**d**,**d’**) C16Br.

The different organoclays exhibited similar features, with an intense resonance band at 33 ppm, indicating that the intercalated surfactants exhibited ordered conformation, with a significant degree of *trans* conformation. Similar data were reported for other organoclays [33,91] (Figure 7). The packing and the conformation of the surfactants were reported to be affected by the amount of C16 intercalation and its orientation between the clay sheets, indeed, *trans* conformation occurred for higher organic contents above the CEC values [33,47,86]. In this study, even though, the content of C_16_ cations was close to the CEC value, the bilayer orientation of the C_16_ cations, lying parallel to the clay sheets made the *trans* conformation more possible. Some degree of gauche conformation was observed only for C_16_Br-CN, and could be caused by the C_16_ cations adsorbed on the external surface of the clay sheets, as indicated by CHN and XRD data.

#### 2.1.5. TGA Data

Another useful technique for the characterization of the organoclays is the thermal analysis technique (TGA). This method examines the thermal stability and thermal decomposition mechanism of the modified clays [92]. In addition, it could be used to estimate the organic contents in the modified organoclays [93,94].

The maximum rate decomposition temperature was determined using the first-order derivative of weight loss-temperature plot. The thermal decomposition expressed in terms of mass loss as a function of temperature (DTG). TGA and differential thermal (DTG) features of pristine clay were divided in two steps, one in the range of 25 °C to 150 °C, associated with a peak in DTG curve at 75 °C, attributed to the mass loss of free water molecules and interlayer water, and the second one was detected in the range of 500 °C to 700 °C with a peak of maximum loss mass at 660 °C, related to structural water (bonded OH that underwent dehydration, Figure 7). The TGA feature was close to those reported in the literature for similar clay types [85,95]

However, the TGA and DTG curves of organoclays exhibited additional mass losses as presented in Figure 7. The first part corresponds to free water region in the temperatures below 100 °C, with a maximum temperature mass loss at 60 °C. The peak shifted to low temperature range, and the decrease in intensity of the corresponding peak was related to the conversion of the environment towards the organophilic of the organoclays [96,97] The second mass loss occurred as the organic surfactants were evolved in the temperature range 150–400 °C, and was a characteristic of defragmentation/oxidation of surfactant cations, which form different arrangements. The third step could be associated to the organic carbon that reacted with inorganic oxygen (combustion reaction) in the range of 570 to 700 °C [97]. The temperature of this peak shifted to lower temperatures as reported in previous studies [33,97,98], and was a consequence of the expansion of the aluminosilicate framework and the resultant easy disconnection of hydroxyl groups from the structural skeleton of host clay mineral [98].

The cloisite Na (CN) did not undergo thermally induced changes in the temperature range of 150 to 600 °C. Thus, the mass loss in this temperature range was attributed to the presence of C_16_ cations in the organoclays. TGA features of the organoclays prepared from different solutions were similar in shape, indicating similar decomposition process of organic surfactants occurred. In comparison to solid C_16_Br salt, the presence of the clays sheets affected its decomposition process, due to the intrinsic effect of the clay sheets, and the maximum temperature loss shifted to higher temperatures [33].

Cloisite-Na is hydrated due to hydrophilic internal surface, and attained a total mass loss of 13%. The dehydration peaks appeared smaller in the DTG curves of organoclays, and the corresponding mass loss percentages at temperatures below 100 °C, was about 3.5%, 3% and 2.7%, respectively. The decrease of mass loss was due to the exchange of Na cations by the C16 surfactants and to the oroganophilic character of the organoclays. The loss of organic surfactants occurred mainly in one step as indicated by the DTG curves with shift of the maximum decomposition temperature from 260 °C to 270 °C, for the C_16_OH-CN sample. This effect could be related to the low value of the basal spacing, that made difficult to lose the organic surfactants (Table 4).

The thermal stability of C_16_-organoclays depended on whether the concentration of surfactant cations was below or above the cation exchange capacity of the clay minerals. In the former case, the high temperatures of surfactant oxidation and mineral dehydration resulted from the ionic interaction between the clay sheets and surfactant forming monolayers in the interlayer gallery [97], whereas in the latter case these evidently lower temperatures are a reflection of predominantly physical sorption of surfactant forming bilayers in the interior and on the surface of the mineral [99,100]. In the present stage, the first explanation was the most plausible.

To calculate the amount of C16 intercalated in organoclays, different methods were proposed by different authors, some of them have taken in account the mass loss of the pristine clay in the studied range of temperature, and others without taking in account such mass loss [101,102,103,104]. The different models were used, and there was a difference between the estimated amounts of C_16_ cations. However, the highest amount of C_16_ surfactants was calculated for C_16_Br-CN clay and the lowest was for the C_16_OH-CN. The C_16_Cl-CN exhibited an intermediate value, in good agreement with the C.H.N elemental analysis data.

#### 2.1.6. Nitrogen Adsorption

The nitrogen adsorption experiments were used to support the existence of C_16_ cations between the interlayer spacing of cloisite Na clay. The cationic surfactant head groups carry positive charge and are tightly bonded to the clay surfaces. Consequently, all cationic surfactants are expected to cover some/all of the mineral surface and decrease the apparent surface area of the surfactant/clay hybrid [105]. The isotherms exhibited type II forms, corresponding to no porous materials, and indicated that the adsorbed amount of nitrogen gas decreased when the C_16_ cations were intercalated in the organoclays. The adsorbed values at higher relative pressures higher than 0.95, was due to the condensation of nitrogen molecules in the interparticle spacing. Besides, cationic surfactant head groups reduce the inter-particle repulsive forces, can cause particles to aggregate, and therefore can also reduce the surface area [106,107]. The S_BET_ of cloisite Na was estimated to 24.6 m^2^/g. this value was in the reported range of similar clay minerals [108] and lower in some cases to the reported ones [109,110]. After intercalation of C_16_ cations, the S_BET_ values of the resulting organoclays decreased and reached a value of 5 to 9 m^2^/g. close to that reported for similar materials [110,111]. These data indicated that the expansion of the basal spacing with organoclays did not lead to an improvement of the surface areas as reported by [112]. This fact could be related to the orientation of the C_16_ cations that lay parallel to the clay layers and thus blocked the passage of N_2_ molecules, occupying active clay sites which might be available for N_2_ molecules. [112,113,114,115,116]. In addition, the pore volume was decreased, due to the presence of the surfactants in the pores available between the clay particles [117]. These surfactants were adsorbed on the exterior surface of the organoclays, as reported in the CHN and XRD paragraphs.

#### 2.1.7. SEM Studies

**Figure 8:**
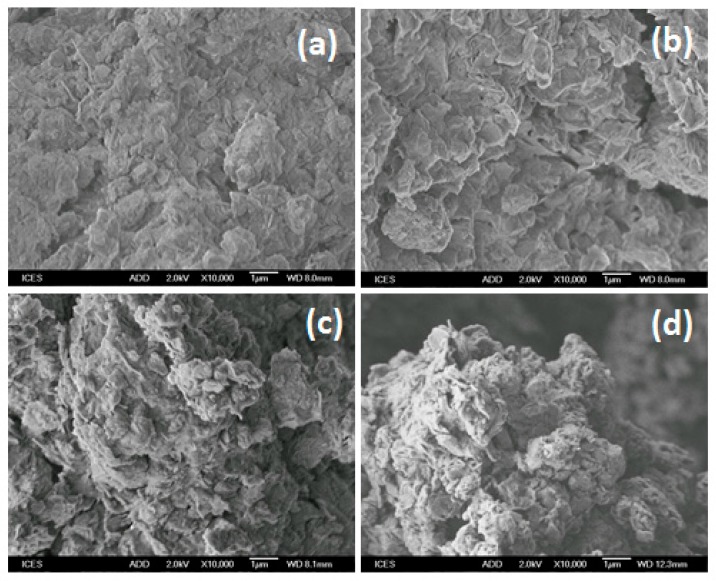
SEM micrographs of (**a**) cloisite Na exchanged with C_16_ solutions. (**b**) C_16_Br, (**c**) C_16_Cl, and (**d**) C_16_OH solutions.

Typically SEM can provide images at micron scale. The SEM micrographs for cloisite Na and organoclay derivative are presented in Figure 8. The particles of the parent clay were agglomerated and compact, while, the particles of the organophilic cloisites were less compact than those of the Na cloisite. The micrographs do not permit us to conclude whether the expansion of the layers is uniform in the whole mass of the modified clay, the morphology of organoclays was moderately affected by the presence of surfactant cations.

The EDX analysis of cloisite Na indicates significant Si (67% in atomic weight) and Al (20.88% in atomic weight) are present with a percentage of Na about 5.22%, indicating the Na character of the cloisite clay. After reaction with C_16_ surfactant solutions, the Na percentage decreased significantly, indicating that most Na cations have been exchanged by C_16_ cations, and were eliminated during the filtration and washing process.

#### 2.1.8. Thermal Stability

The organoclays were used to remove acid dyes from polluted water, in this part, the in-situ XRD was used to investigate the structural changes of the organoclays and to identify the appropriate temperature at which the organoclays could be thermally treated without losing their performance in the eosin removal process [29,33].

For comparison, the thermal stability of the C_16_Br salt used was investigated. Previous studies indicated that the solid C_16_Br exhibited a layered structure with a length of the C_16_ chain about 2.6 nm, this value was close to that reported in the literature [24,116]. By heating the salt, the in-situ the PXRD study indicated that the layered structure expanded from 2.60 nm to 3.32 nm, in the temperature range of 25 °C to 210 °C, then it collapsed, due the melting of the solid salt [33,47] (Supplementary Material 3).

**Figure 9:**
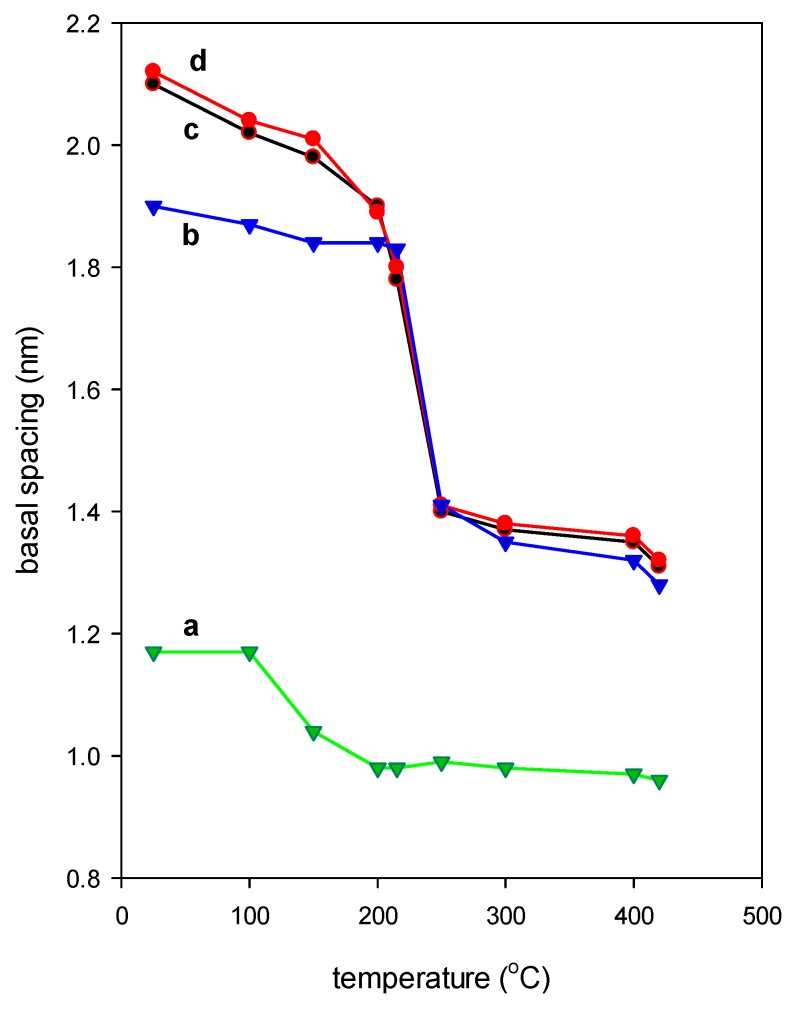
Variation of the basal spacing of starting (**a**) cloisite-Na clay and derived organoclays (**b**) C_16_OH-CN, (**c**) C_16_Br-CN, and (**d**) C_16_Cl-CN preheated at different temperatures.

The in-situ PXRD of the C_16_Cl-CN precursor showed that the basal spacing of 2.00 nm was maintained at temperatures up to 150 °C, and indicating that the removal of water molecules did not affect the layered expansion, and the water molecules were mainly adsorbed onto the external surface of the organoclays, as described in the TGA section. The basal spacing of 2.10 nm decreased slightly in this range, and it varied from 2.10 to 1.95 nm (Figure 9). At a temperature of 250 °C, a dramatic decrease of the basal spacing occurred from 1.95 nm to 1.41 nm. This temperature value coincided with the maximum temperature loss in the DTG curve. The decrease could be related to the release of one layer of C_16_ cations, and the value of 1.41 nm was close to an intercalated monolayer of C_16_ cations between the clay sheets. The basal spacing of 1.41 nm decreased continuously from 1.40 nm to 1.35 nm, indicating that the C_16_ cations were not completely destroyed at the heated temperatures. The maximum in-situ temperature was 420 °C, and no further change was observed due to the limitation of the operational temperature.

In general two sets of basal spacinga were observed for the different organoclays, one up to 2.02 nm and the second one started from 1.40 nm due to a possible loss of one monolayer of surfactant cations, or to the presence of residual carbon materials between the clay layers. The intercalated C_16_ cations behave differently than the C_16_Br salt used (Figure 9). This difference could be associated to the confined space of the clay layers, or to the conformation of the C_16_ cations in the bromide salt used. The calcination of the organoclays at temperatures higher than 500 °C led to further decrease of the basal spacing up to 1.28 nm, and indicated the presence of residual carbon materials [47], compared to the value 0.96 nm for pristine clay mineral calcined at 500 °C.

### 2.2. Removal of Eosin Dye

The prepared organoclays and their calcined products were tested in the removal of the acidic dye eosin.

#### 2.2.1. Effect of Initial Concentration

In this part, different initial concentrations (C_i_) were used in the range of 25 ppm to 1000 ppm, for the sample C_16_Br-CN. At a constant amount of organoclay, the removal percentage of eosin decreased from 100% to 58%. as the C_i_ values were varied from 25 ppm to 1000 ppm (Supplementary Material 4). This indicated that the concentration gradient is an important driving force to overcome the mass transfer resistances between the liquid and solid phase [118]. At lower eosin concentrations, the ratio of solute connecting to the organoclay sites is higher, which caused the increase in color removal efficiency, while at higher dye concentration, the lower adsorption percentage was caused by the saturation of active removal sites on the organoclay surface. On the other hand by increasing the eosin initial concentration, the actual amount of eosin removed per unit mass of organoclay increased from 2.5 mg/g to 50 mg/g (Supplementary Material 4). The effect of used C_i_ of eosin was examined for the other two organoclays, and the removed amount was depended on the initial concentration and it was improved from 25 ppm to 1000 ppm with a maximum at 40 to 45 mg/g, for C_16_Cl-CN and C_16_OH-CN materials. The high removal efficiency at lower concentrations may be due to the existence of more available vacant sites on the organoclay than the number of eosin ions existed in the solution. At higher concentrations, the eosin anions are comparatively higher than the available vacant sites for the removal [33,85].

#### 2.2.2. Effect of Surfactant Content

The content of intercalated C_16_ cations were investigated using 0.1 g of solid material and varying the initial concentrations from 25 ppm to 1000 ppm. The starting CN clay exhibited a low removal capacity of about 3 mg/g. This low value was related to the nature of the negatively clay surface, per consequent a repulsion between the surface clay and the eosin anions in solution [23,78]. Similar data were reported for other acidic dyes using different negative charged solids [32,119,120]

When the CN was modified by cationic surfactants, an improvement of the removal capacity was observed, especially at higher C_i_ values. The C_16_Br-CN exhibited the highest removal capacity. This fact was related to its highest content of surfactants as mentioned in Table 1. Meanwhile, C_16_OH-CN exhibited the lowest removal capacity value. The structure of Si-O groups and hydration of Na^+^ ions in the clay establishes a hydrophilic structure on the mineral surface. However, the anionic surface properties of the clay can be changed using positively charged organic compounds such as alkyl ammonium ions. Thus, an improvement of removal efficiency was related to the complete covering of the negative charge on CN clay by the surfactant, which means that the electrical repulsion was overcame [120]. Similar data were achieved using other clay minerals and layered silicates [33,66].

**Figure 10:**
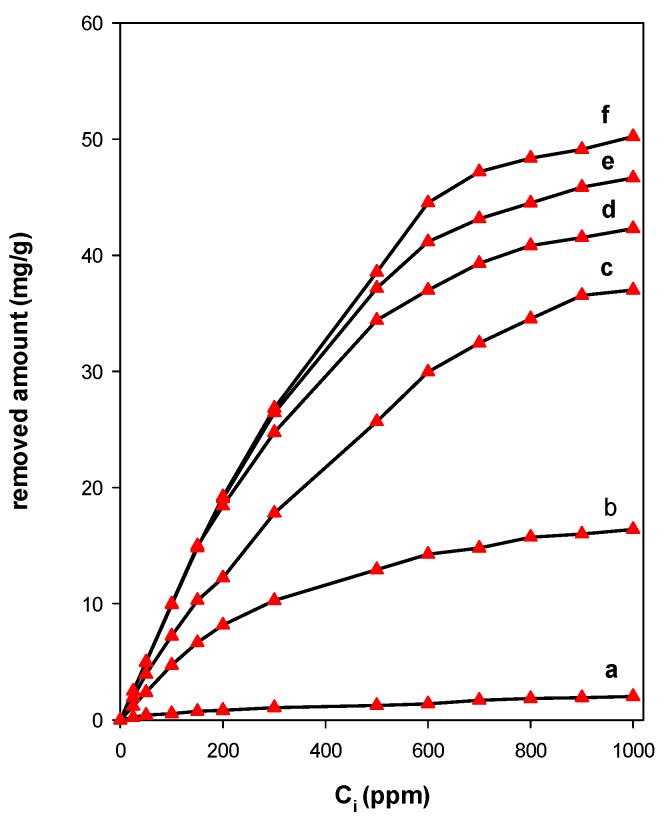
Eosin Removal properties of cloisite-Na loaded with different C_16_ cations, (**a**) 0 mmol/g; (**b**) 0.32 mmol/g. (**c**) 0.54 mmol/g, (**d**) 0.90 mmol/g. (**e**) 0.99 mmol/g, and (**f**) 1.05 mmol/g.

In case of C_16_Cl-CN loaded with different C_16_ contents, similar trends were observed (Figure 10). At lower C_i_ values below 100 ppm, the removed amount was not affected by C_16_ loadings. However, a continuous improvement of eosin removal efficiency was observed, and maximum value of 52 mg/g was obtained for C_i_ value close to 1000 ppm. At intermediate C_16_ loadings below the CEC values, the clay surfaces still exhibited a negative charge character that affected the eosin removal properties. However, at loading close to the CEC value, the negative charge was totally overcame by the C_16_ cations, and thus made easy the removal of the anionic dyes. Previous studies indicated that the accumulation of quaternary ammonium cations in the interlayer and on surfaces largely in excess of the CEC of the clay mineral leads to a build-up of net positive charges. This charge reversal consequently improves the material’s affinity to negatively charged contaminants such as metalloids (e.g., chromate, arsenate) [121] surfactants [122], and herbicides [123].

#### 2.2.3. Effect of Removal Temperature

The removal properties of a selected organoclay (C_16_Br-CN), was investigated at three different temperatures from 25, 40 and 50 °C. The selected value of 50 °C was chosen because the organoclays were stable at this temperature as mentioned above. The effect of the temperature was clearly noticed at C_i_ values greater than 300 ppm. Al lower C_i_ values, a removal of 100% was achieved, independently of the operational temperatures (Supplementary Material 5). However, at higher initial concentration of 1000 ppm, a value of 65 mg/g was achieved at 50 °C. The improvement of the removal efficiency of eosin with an increase in temperature was owed to the strength of the attractive force between the removal sites and eosin, and demonstrates an endothermic process [33].

#### 2.2.4. Effect of Heating Temperature of Organoclays 

**Figure 11:**
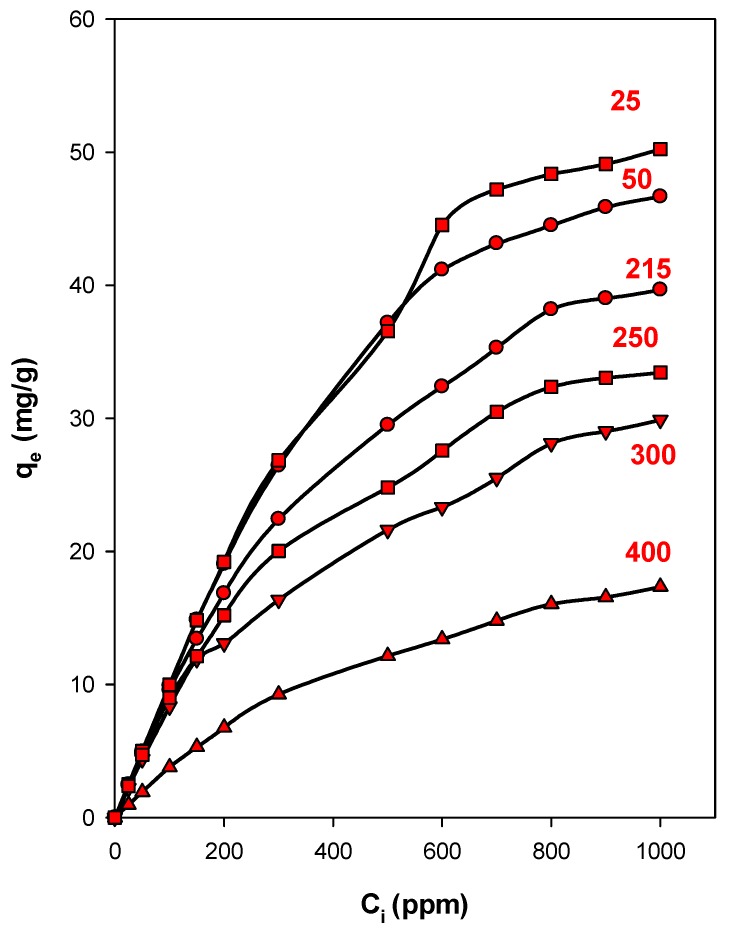
Eosin removal properties of C_16_Cl-CN organoclay preheated at different temperatures (°C).

This study was focused on the C_16_Cl-CN organoclay, and the material was pre-heated at selected temperatures deduced from Figure 9. The C_i_ values of the eosin were varied from 25 ppm to 1000 ppm. The data are presented in Figure 11, and indicate that the removed amount of eosin was independent of the preheated temperatures lower than 215 °C for C_i_ values less than 200 ppm. However, as the preheating temperatures increased, for example above 215 °C, a decrease of the removed amounts was observed for used C_i_ values above 200 ppm. This variation was related to the shrinkage of basal spacing from 1.91 nm to 1.41 nm, and thus reduced accessibility of eosin anions to the removal sites. Interestingly, the removal amounts were comparable to other organoclays with similar basal spacing. As reported previously, the decrease of the basal spacing was related to the decomposition of organic surfactants, as showed by the TGA and in-situ XRD studies, and thus affected the removal properties of the preheated materials. At preheated temperatures of 300 °C, the removal efficiency of derived material was reduced due to the complete destruction of the intercalated surfactants, and to the loss of the removal sites. However, it was higher than the starting CN clay. This difference was attributed to the remaining of the carbon materials between the clay layers. Similar data were reported for different organo-clays and organo-silicates materials, such as magadiite or kenyaites, for the removal of eosin and nitrobenzene [33,53,65,123].

#### 2.2.5. Maximum Removal Amount

The determination of the maximum removal capacity and the development of an equation that could be accurately used for design purpose are important factors for economical purposes. Langmuir model is among the most common isotherm that can be used for the description of solid–liquid sorption system [124].

The well-known expression of the Langmuir model is given in Equation (3):

(3) $$\frac{C_{e}}{q_{e}}=\frac{1}{q_{\max}\cdot K_{L}}+\frac{C_{e}}{q_{\max}}$$

where *q_e_* (mg/g) and *C_e_* (mg/L) are the amounts of adsorbed dye per unit weight of adsorbent and unadsorbed dye concentration in solution at equilibrium, respectively. *q*_max_ (mg/g) and *K_L_* (L/mg) are the Langmuir constants, representing the maximum adsorption capacity for the solid phase loading and the energy constant related to the heat of adsorption respectively

The data are presented in Table 5. The application of the Langmuir model to the adsorption isotherm showed that the Langmuir isotherm model turned out to be extremely satisfactory with higher R^2^ value greater than 0.99.

The results indicated that the modification of cloisite clay by surfactant cations improved its eosin removal properties compared to the starting clay. In overall, the organoclays exhibited close to maximum removal amounts varying from 46 to 55 mg/g of clay. This fact could be related to the amount of intercalated C_16_ cations. From the values in Table 5, the surface area and pore volume were not important factors in terms of controlling the affinity between organoclays and dye anions [116], and the loaded surfactant was highly important for determining the removal capacity of the organoclays.

The pre-heating of the organoclay also affected the removal properties, and an average value of 47 mg/g was maintained at temperatures below 210 °C, then it dramatically decreased to 30 mg/g after that temperature. This fact was related to the destruction of the intercalated C_16_ cations, and thus the unavailability of removal sites to this process. These data confirmed the idea that the surfactant content was the most important factor for the determination of the removal capacity.

The Langmuir constants were related to the affinity of the surface clay towards the eosin anions, and in overall, these values were improved as the removal amount increased, in good agreement with the previous studies [33,67].

In comparison to other organoclays and adsorbents (Table 6), the organoclays prepared from cloisite exhibited reasonable removal properties and could be used for temperatures less than 200 °C without significant loss of their capacities. In comparison to organo-polymer grade montmorillonites (organo-PG clays), the later exhibited higher removal capacities than the organocloisites, this fact was related to the higher CEC of the polymer grade (PG) clay (about 1.40 meq/g) [33], per consequent, a higher up take amount of C_16_ cations was achieved. In case of magadiite, it exhibited a large CEC value above 2 meq/g [53], nevertheless, its uptake of C_16_ cations was lower than that of the PG clay. The magadiite was prepared in the laboratory and it was difficult to exfoliate it in pure dionized water, thus, the up take amount was limited and close to CEC value, due to the cation exchange reaction between the Na^+^ cations and the C16 ones. In case of cloisite-Na clay, the challenging problem consists of improving the up take amount of C_16_ cations, above the CEC value. This target will be investigated separately.

### 2.3. Regeneration Tests

**Figure 12:**
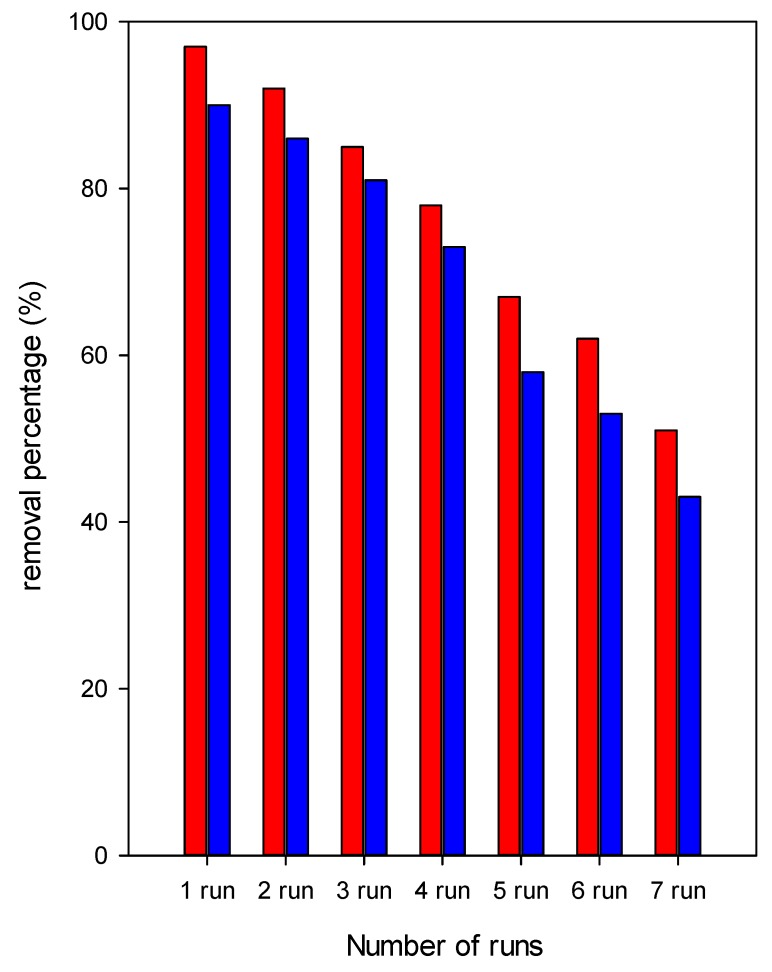
Regeneration properties of C_16_Br-CN (red) and C_16_OH-CN (blue) organoclays.

The processes of regeneration and reuse of these clay materials are of high interest; a cost-effective and feasible regeneration methods could be developed to make use of the organoclays with real industrial dye effluents economically viable [130]. In this case, a regeneration process based on the oxidation reaction of the removed dye was adopted, using a minimum of oxidant agent and chemicals [33]. This method was reported to be effective and easy to use. Two samples were selected, the C_16_Br-CN and C_16_OH-CN clays. The data are presented in Figure 12 and it can be seen that the removal capacity of the decreased continuously with an increase in the number of regenerations of the used organoclays, during the first four runs, a decrease of about 20% was obtained for C_16_Br-CN and of 30% for C_16_OH-CN. After the fifth run, C_16_OH-CN material exhibited a significant reduction above 50%, however, the C_16_Br-CN still maintained a reasonable 65% removal. The decrease in the eosin removal efficiency suggested that the availability of the number of removal sites decreased with the increase in numbers of runs on the organoclays, and could indicated that the removed eosin anions were strongly attached to removal sites and thus were difficult to be removed during the regeneration process. In previous study, the regeneration efficiency was related to the C16 contents in the organoclays. Organoclays with higher C_16_ contents exhibited a slight decrease after four runs of 20%, and still maintained a removal efficiency of 70% after five reuse cycles [34].

### 2.4. Alternative Approach

The removal agent cost is important parameter for comparing the industrial applicability of materials. The overall cost of the adsorbent is governed by several factors, including its availability, the processing required, and its reuse. The clay mineral is available in abundance everywhere, near the local communities. The transformation of the raw clay mineral to homoionic derivative is not compulsory, and it saves water and chemical consumptions. In mean time, the modification of clay mineral by alkyl ammonium cations will add extra processing cost. This cost will be compensated by its better pollutant removal efficiency. In addition, the preparation of organoclays requires a much larger amount of water and surfactants, and results in much larger quantity of wastewater and surfactant with excess inorganic quaternary ammonium anions. Thus, a balance between a proper amount of added water and surfactant in the preparation of organoclays should be established. Solid state intercalation is easy to perform and is one of the most suitable techniques for intercalation processing. This technique, which consists of grinding a clay mineral and a dry surfactant, at room temperature for a short period of time could be used. Some literature indicates that the surfactants can exhibit significant harmful effects to living organisms including bacteria, protists and animals. However, once intercalated into clay minerals, the surfactants will not be released into the water, and materials containing organoclays have almost zero leaching, making them completely safe to use outdoors, with no or low impact on the environment.

## 3. Experimental Section

### 3.1. Materials

**Figure 13:**
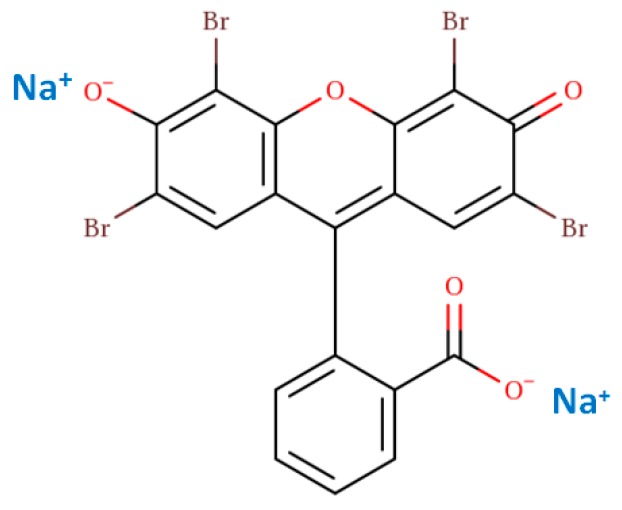
The chemical structure of the used eosin dye.

The starting cloisite clay (CN) was supplied by Nanocor Company (city, state abbrev USA) with a cation exchange capacity (CEC) of 95 meq/100 g, as reported by the supplier. The clay was used as received [131]. Hexadecyltrimethyl ammonium bromide (C_16_Br), hexadecyltrimethyl ammonium chloride (C_16_Cl), and hexadecyltrimethyl ammonium hydroxide (C_16_OH) were purchased from TCI Chemical Company (TCI, Singapore), they were of analytical grade. Eosin (99.9%) was purchased from Acros Organics (Loughborough, UK). The structure is presented in Figure 13. Oxone (2KHSO_5_×KHSO_4_×K_2_SO_4_, 4.7% active oxygen) and cobalt nitrate hexahydrate were purchased from Alfa Chemicals (Binfield, Berkshire, UK). All the chemicals were used as received.

### 3.2. Modification of Organo-Clays

The organocloisite was prepared by a cation exchange reaction as reported in a previous study [33]. In a typical preparation, a known amount of C_16_Br salt (corresponds to 2.44 mmole) was dissolved into 50 mL of deionized water, then two grams of cloisite clay (CN) were added to this solution. The dispersion was stirred overnight at room temperature. The resulting material was separated by filtration, and washed repeatedly with deionized water for 6 to 7 times. The sample was dried at 40 °C for overnight in a statistical oven.

When C_16_ solution was used with different anions such chloride and hydroxide, and at same concentration of 2.40 mmole, the corresponding mass of the surfactants was dissolved into 50 mL of deionized water, and then similar procedure was followed as for the C_16_Br salt.

The sample will be identified as C_16_X-CN, where X corresponds to the used anion of C_16_ salt. For example, C_16_Br-CN indicates that cetyltrimethylammonium Br salt was used to prepare the organoclay.

### 3.3. Effect of Washing Solution

This study concerns the C_16_Br-CN organoclay, after its preparation as reported in Section 3.2, and during the filtration process, ethanolic solutions with different ratios of ethanol to water (% in volume) were used, instead of pure deionized water. The samples were washed several times with the solution, then dried at room temperature [33].

### 3.4. Chemical Stability

The C_16_Br-CN was used as a model sample, and the experiments were conducted as reported in previous study. 50 mL of aqueous solutions of NaCl, HCl and NaOH with concentrations of 0.5 M were prepared, then 0.5 g of solid C_16_Br-CN were added to each solution, separately. The suspension was stirred for overnight, the solids were collected by filtration, washed with deionized water for several times, then dried at room temperature [33].

### 3.5. Eosin Removal

The removal experiments of eosin dye were performed as reported previously [54]. A stock solution of 1000 mg/L was prepared by dissolving 1 g of eosin dye into 1000 mL of deionized water. The dilution process was carried out to get the desired concentrations. 100 mg of solid materials were added to 10 mL of different initial concentrations (50 to 1000 mg/L) of eosin in separated glass vials of 12 mL capacity. The sealed vials were shacked into on a water-bath shaker at fixed temperature of 25 °C and for overnight, to ensure that the equilibrium was attained. The solutions were centrifuged at 4000 rpm for 10 min, and the residual concentrations of eosin in the filtrates were determined from the calibration curve at absorbance maxima, λ_max_ of 516 nm. The effect of temperature on the eosin removal efficiency was performed at different temperatures from 25 °C to 50 °C, following the same procedure described above.

Blank experiments were performed using neat dye solutions (without solids) to ensure that no dye was adsorbed onto the glass tubes. All removal experiments were performed in duplicate and the mean values were used in data analysis, the percentages of the errors were about 5%. The methods to estimate the amount of removed eosin (*q_e_*, in mg/g) and the removal efficiency (%) of the dye at equilibrium were reported in previous study [33].

### 3.6. Regeneration Process

Regeneration studies on the spent organoclays were studied to determine possible reusability of the organoclays after batch adsorption experiment. Organoclay was first added to 50 mL of eosin (200 mg/L) at 25 °C. The eosin-loaded organoclay was separated by centrifugation, washed with water and then treated into 10 mL of a cobalt nitrate solution of (10 mM) and 12 mg of oxone, for 30 min. The oxone was added into the mixture to degrade the removed eosin [33,48]. The regeneration process was repeated for seven cycles, following the same procedure.

### 3.7. Characterization

CHN elemental analysis was performed using an EURO EA elemental analyser (Waltham, MA, USA) for the different organoclays. Two parallel runs for each sample were performed. Powder X-ray diffraction (PXRD) patterns were collected on a D8 Advance powder diffractometer (Bruker, Germany) using monochromatic Cu Kα radiation (λ = 0.15406 nm). Fourier-transform infrared (FTIR) spectra of the samples were recorded by KBr disk method on a Shimadzu FTIR spectrometer (Tokyo, Japan) over the spectral region of 400–4000 cm^−1^. Thermo-Gravimetric Analysis (TGA) was performed on a model SDT2960 TA instrument (New Castle, DE, USA solid ^13^C-CP-NMR technique was performed to investigate the conformation of the intercalated C16 cations. The detail analysis was reported in a previous work [33]. The morphology was observed by a model JSM-6700F field scanning electron microscope (Jeol, Japan) equipped with an EDX system. N_2_ adsorption isotherms were collected at 77 K on a ASAP 2040 system (Micromeritics, Ottawa, ON, Canada) and the pore volume was estimated at a relative pressure of P/P_o_ at 0.95. The specific surface area was determined by the Brunauer–Emmett–Teller (BET) method. The samples were degassed to 120 °C for overnight.

The powder in-situ x-ray diffraction patterns were collected using a HTK 16 high temperature chamber (Anton Paar, Ostfildern-Scharnhausen, Germany) mounted on a Bruker AXS, D8 Advance diffractometer [54]. The temperature was varied in the range of 25 °C to 420 °C. A UV-VIS spectrophotometer (Cary 100 model, Varian, Australia) was used to estimate the absorbance at maximum wavelength (λ_max_ = 610 nm) in the supernatant, and the concentration at equilibrium was estimated from the calibration curve.

## 4. Conclusions

The removal capacity by cloisite Na clay was improved to a huge extent due to the organic modification by cetyltrimethylammonium cations, Actually, the C_16_ cations impart organophilicity to the starting cloisite, due to cation exchange of Na cations by the surfactant cations. As a result, the resulting organocloisites removed more eosin than the unmodified clay. The removal amount was related to the content of the C_16_ surfactants, initial concentrations of eosin, and the preheating temperature of the organoclay prior its use.

Overall, the type of the anion used for the surfactant salt did not significantly affect its intercalated amount, and the values were in the range of 0.95 mmol/g to 1.04 mmol/g, with an expansion of the basal spacing that it did not exceed 2.05 nm, and associated with a bilayer arrangement of C_16_ cations parallel to the clay clays. The preheat treatment of two selected OCs affected their eosin removal properties at temperatures higher than 215 °C. The regeneration process indicated that the removal property was maintained up to four cycles, depending of the used organoclays. The prepared organoclays can be safely used with low or no impact on environment. Since, the intercalated cations were not exchanged with smaller cations such as Na or protons for a longer contact time with the corresponding solutions, compared to their quaternary ammonium salts on their owns.

## Figures and Tables

**Table 1:** CHN elemental analysis of different OCs prepared from different solutions.

Samples	C%	H%	N%	C/N *	Uptake Amount (mmole/g) ^+^
C_16_Br salt	62.62	11.67	3.87	18.87	-
C_16_BrCN-2.40	28.12	4.63	1.80		1.23 (1.44)
C_16_ClCN-2.40	20.47	4.36	1.30		0.90 (1.05)
C_16_OHCN-2.40	18.56	3.60	1.16		0.81(0.95)

* molar ratio; + Uptake amount = C(%)/[(12 × (number of carbon atom in CTMA)] × 1000; - Not applicable.

**Table 2:** C, H, N elemental analysis of different C_16_Cl-CN prepared from C_16_Cl solutions with different initial loadings.

Samples	C%	H%	N%	C/N *	Uptake Amount (mmol/g) ^+^
C_16_ClCN-0.41	7.21	2.48	0.46	18.28	0.32 (0.34)
C_16_ClCN-0.83	12.29	2.65	0.76	18.82	0.54 (0.64)
C_16_ClCN-1.20	17.66	3.78	1.09	18.90	0.77 (0.91)
C_16_ClCN-2.40	20.47	4.36	1.27	18.80	0.90 (1.05)
C_16_ClCN-3.30	22.63	4.64	1.41	18.33	0.99 (1.16)
C_16_ClCN-4.80	23.79	4.81	1.47	18.88	1.04 (1.22)

* molar ratio; + Uptake amount = C(%)/[(12 × (number of carbon atoms in C_16_)] × 1000.

**Table 3:** Solid ^13^C-CP-NMR resonance signals and their assignments for the C_16_ surfactant used.

Sample	Spectral Assignment (Shift in ppm)	Structure
Solid C16Br [88]	C1: 68; C2: 32; C3-C14: 30; C15: 27, C16: 24; C17-C19: 54	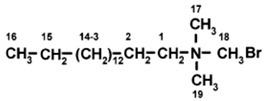
Solid C16Cl [89]	C2: 67.05 (66.80) *; C1: 54.61 (53.14); C15: 36.40 (31.92); C5-C14: 34.70 (29.44); C3: 30.77.29.19 (26.24); C4:27-24 (23.25); C16: 27-24 (22.68); C17: 18.22, 17.10, 16.56, 16.14 (14.12)	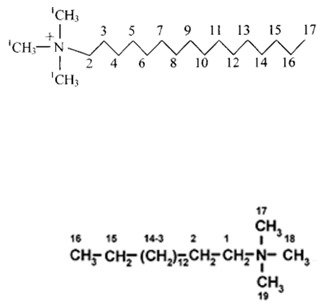
C16Cl-CN	C17: 14.9; 23.7 (C16); 33.2 (C15); 27.4 (C3); 67.8 (*N*-methyl group (C1); 31.1 (C4-C14)	

* Values between brackets (in solution).

**Table 4:** Thermal properties of cloisite and derived organoclays.

Samples	T_max_ (°C) of H_2_O Molecules	T_max_ (°C) of C_16_^+^	T_max_ (°C) of Residual C_16_ and Dehydration	W (%)
CN	75	-	690	85.5
C16Br-CN	54	260	580	60.77
C16Cl-CN	45	260	560	70.95
C16OH-CN	54	270	590	72.91
C16Br	-	242	-	1

T_max_: maximum rate decomposition temperature; W: remaining mass after heating at 900 °C; - non applicable.

**Table 5:** Langmuir parameters of eosin removal by the different organoclays.

Samples	q_max_ (mg/g)	K_L_ (L/mg)	R^2^
CN	2.25	0.0035	0.9343
C_16_Br-CN	55.64	0.1332	0.9979
C_16_Cl-CN	50.50	0.0844	0.9956
C_16_OH-CN	46.66	0.0773	0.9954
C_16_Cl-CN-50	50.17	0.0459	0.9915
C_16_Cl-CN-150	50.02	0.0371	0.9945
C_16_Cl-CN-200	47.56	0.0268	0.9954
C_16_Cl-CN-215	40.70	0.0182	0.9953
C_16_Cl-CN-250	31.41	0.0135	0.9942
C_16_Cl-CN-300	29.42	0.0076	0.9912
C_16_Cl-CN-400	24.72	0.00275	0.9902
C_16_Cl(0.32)*-CN	19.98	0.00542	0.9932
C_16_Cl(0.54)-CN	42.31	0.0621	0.9943
C_16_Cl(0.9)-CN	46.23	0.0684	0.9903
C_16_Cl(1.05)-CN	46.85 50.50	0.0909 0.0844	0.9934 0.9959

* uptake of C_16_ cations.

**Table 6:** Removal capacities of various adsorbents for eosin dye.

Samples	q_m_ (mg/g)	References
Organo-CN clays	34.96 to 51.81	This study
Organo-PG clays	75.11 to 94.20	[33]
Organo-magadiites	69.54	[53]
Organo-local clays	48.66	[125]
Organo-kenyaites	48.01	[66]
5Diethylentriamine-montmorillonite	11.90	[126]
Raw fly ash	43.48	[127]
Alumina nanoparticles	47.78	[128]
Teak leaf litter powder	31.64	[129]

## Supplementary Materials

The following are available online.
