# Supplementary material for: Eosin Removal by Cetyl Trimethylammonium-Cloisites: Influence of the Surfactant Solution Type and Regeneration Properties
Source: Molecules. 2019 Aug 20;24(16):3015. doi: 10.3390/molecules24163015 (PMC6720785; doi:10.3390/molecules24163015)
Supplement: Supplementary file 1 [file molecules-24-03015-s001.pdf]

Supplementary information of submitted manuscript: " Eosin Removal of Cetyl trimethylammonium-Cloisites: Influence of the surfactant solution type and regeneration properties"

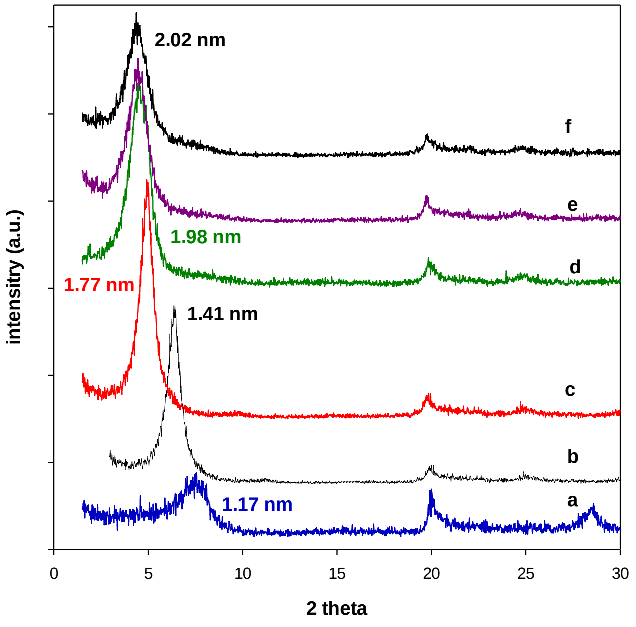

Suplementary material 1. Powder XRD patterns of (a) cloisite clay exchanged with C16Cl solution using different concentrations (b) 0.20 mM, (c) 0.40 mM; (d) 1.20 mM, (e) 1.60 mM, and (f) 3.20 mM.

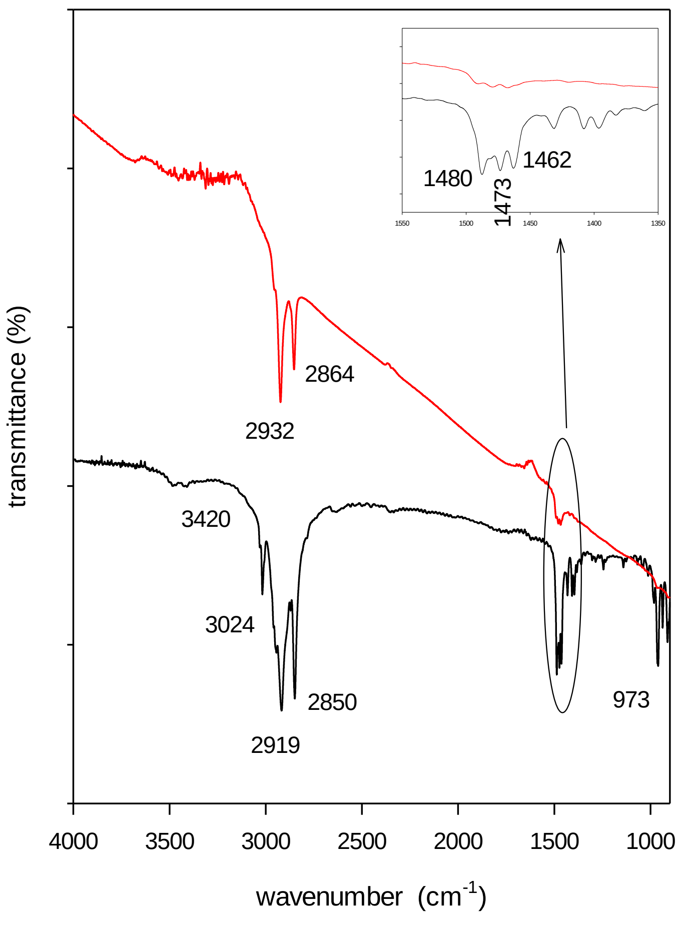

Supplementary material 2. FTIR spectra of solid C16TMABr salt (black) and liquid C16TMABr (red)

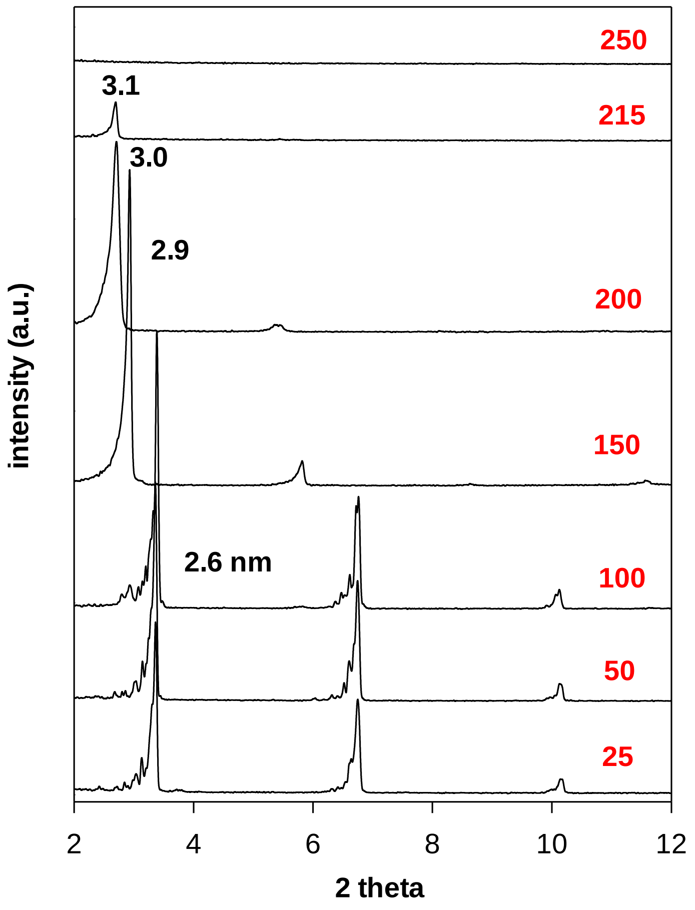

Supplementary material 3. *in-situ* Powder XRD patterns of C16TMABr salt preheated at different temperatures (^{o}C).

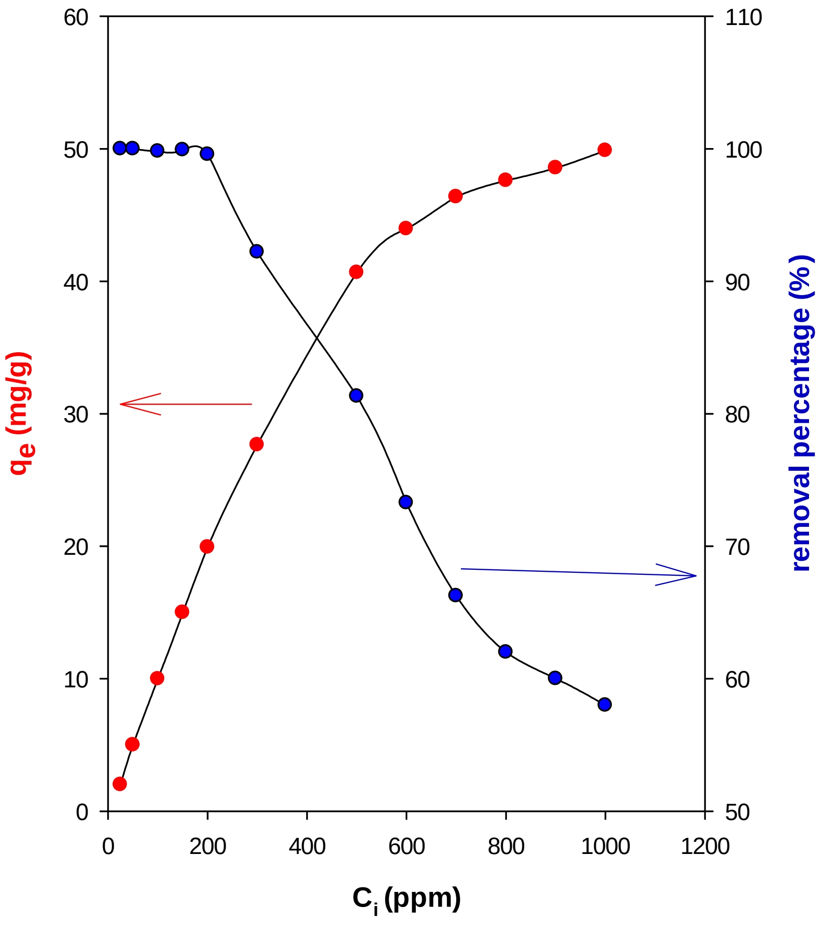

Supplementary material 4. Removal percentage (R%, red) and removal amount (q_e, blue) of C16Br-CN material.

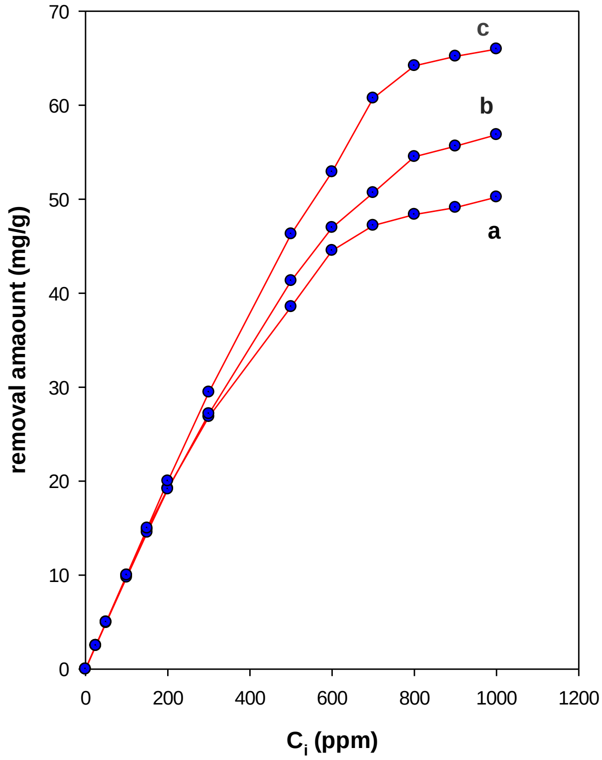

Supplementary material 5. Eosin removal properties of C16Cl-CN at different temperatures (a) 25 ^{o}C, (b) 35 ^{o}C, and (c) 50 ^{o}C.
